# Surgical repair of traumatic right subclavian artery pseudoaneurysm after war shrapnel injury: A case report

**DOI:** 10.1016/j.jvscit.2026.102381

**Published:** 2026-06-29

**Authors:** Ghadeer Ayash, Anas abdulrazzak, Lamis Aroos, Younes Souleiman, Ammar Mohammad

**Affiliations:** a Departments of Vascular Surgery, National University Hospital, University of Damascus, Damascus, Syria; b Thoracic Surgery, National University Hospital, University of Damascus, Damascus, Syria; c Anesthesiology, National University Hospital, University of Damascus, Damascus, Syria; d University of Damascus; and the Departments of Thoracic Surgery, National University Hospital, University of Damascus, Damascus, Syria; e Vascular Surgery, National University Hospital, University of Damascus, Damascus, Syria

**Keywords:** Subclavian artery, Pseudoaneurysm, Vascular trauma, Surgical repair, War injuries

## Abstract

A rare right subclavian artery pseudoaneurysm developed after a war-related shrapnel injury in a 46-year-old female with a prior thyroidectomy. There was no initial bleeding or neurologic deficit, and initial noncontrast computed tomography findings were negative. One month later, the patient presented with chest pain, hoarseness, and dysphagia. A chest X-ray showed tracheal compression, Doppler ultrasound revealed leakage from the posterior wall of the right subclavian artery, and computed tomography angiography confirmed a 6 × 7 cm pseudoaneurysm causing 70% tracheal narrowing. Open repair was performed via partial sternotomy and a subclavicular incision using a 7-mm Dacron graft. The hospital stay was 3 days (1 day in the intensive care unit), without complications. Follow-up Doppler at 1 and 3 months showed graft patency, normal palmar arch waveforms, and symptom resolution.

Traumatic pseudoaneurysm of the right subclavian artery is an exceedingly rare condition, representing fewer than 0.1% of hospital admissions because of traumatic injuries and approximately 1% to 2% of traumatic vascular injuries.^1^, ^2^, ^3^ War shrapnel, small, high-velocity metallic fragments produced by shell or improvised explosive device explosions, causes blast-related arterial wall disruption without necessarily producing a large external wound. No precise incidence rate for shrapnel-induced subclavian artery pseudoaneurysm is available because of its extreme rarity.^4^ A pseudoaneurysm results from disruption of the arterial wall, with bleeding contained by the surrounding tissues. This condition exposes patients to risks of life-threatening hemorrhage, brachial plexus compression, or rapid enlargement. Presentation may be delayed for weeks or even months, which complicates early diagnosis and management.

Peripheral artery injuries in war settings are more commonly reported in the axillary (26.7%), brachial (20%), and popliteal (13.3%) arteries, whereas subclavian artery pseudoaneurysm after war-related shrapnel injury is considered extremely rare.

This report presents an additional case of traumatic right subclavian artery pseudoaneurysm following war shrapnel injury, emphasizing diagnostic nuances and outcomes in a penetrating war-trauma setting.

## Case presentation

A 46-year-old female with a surgical history of thyroidectomy at age 13 years and lifelong levothyroxine replacement therapy sustained a war shrapnel injury to the right supraclavicular region. The entry wound was located above the right clavicle, with no obvious exit wound. The patient reported being in close proximity to a mortar shell explosion, and a small, high-velocity metallic shrapnel fragment caused the injury. At the initial presentation, she exhibited no signs of active external hemorrhage, expanding hematoma, or neurologic deficit. Management included 24-hour observation with hemodynamic monitoring and a noncontrast chest computed tomography (CT), which showed no evidence of active vascular injury. The CT scan identified a metallic shrapnel fragment measuring approximately 6 × 8 mm, located adjacent to the esophagus and the trachea (Fig 1).

**Fig 1:**
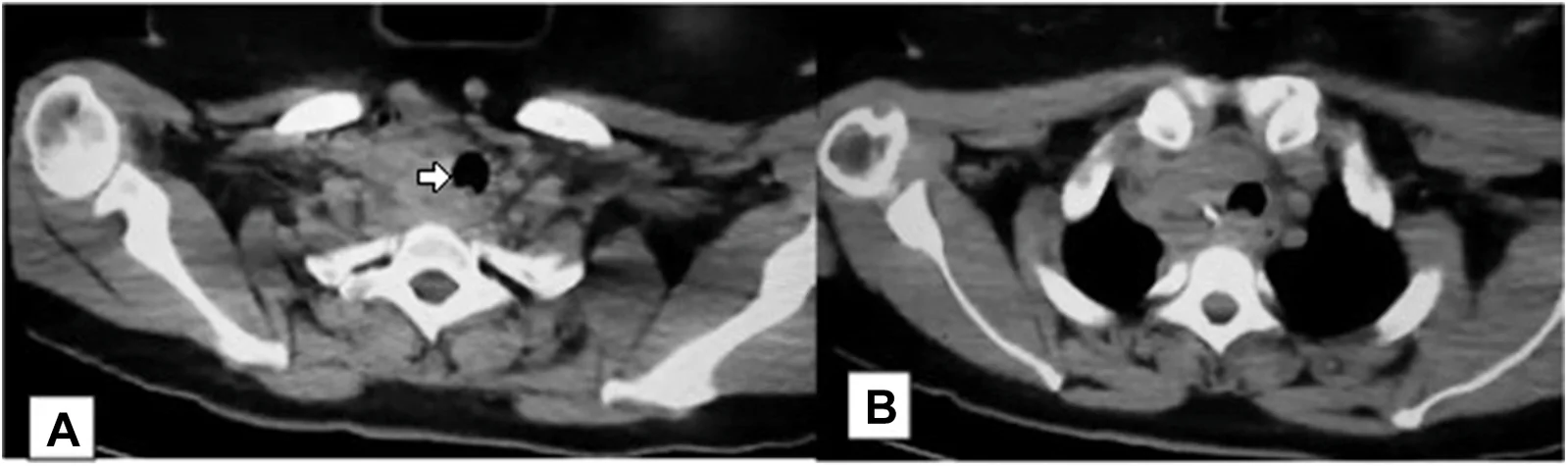
Initial noncontrast chest computed tomography (*CT*) scan. Axial view **(A)** and **(B)** demonstrates a 6 × 8-mm metallic shrapnel fragment located adjacent to the esophagus and trachea in the right supraclavicular region. Mild tracheal deviation is noted (*white arrow*). No evidence of active vascular injury or hematoma is observed at the time of initial presentation.

Computed tomography angiography (CTA) was not performed at initial presentation because the patient was hemodynamically stable and had no thrill or bruit, expanding hematoma, or clinical signs of acute vascular injury. Endoscopy was not indicated as the patient reported no dysphagia, odynophagia, or esophageal symptoms.

One month later, the patient developed right-sided chest pain, accompanied by severe hoarseness and dysphagia. Plain chest radiography revealed a mass effect compressing the trachea. A noncontrast chest CT was subsequently performed, which demonstrated a mass lesion suggesting several differential diagnoses, including hematoma, substernal goiter, and pseudoaneurysm (Fig 2). Doppler ultrasound demonstrated leakage from the posterior wall of the right subclavian artery, proximal to the origin of the right common carotid artery from the brachiocephalic trunk. Multislice contrast-enhanced CT angiography confirmed a 6 × 7-cm pseudoaneurysm arising from an 8 × 9-mm defect in the posterior wall of the right subclavian artery, resulting in approximately 70% tracheal narrowing because of compressive mass effect (Fig 3).

**Fig 2:**
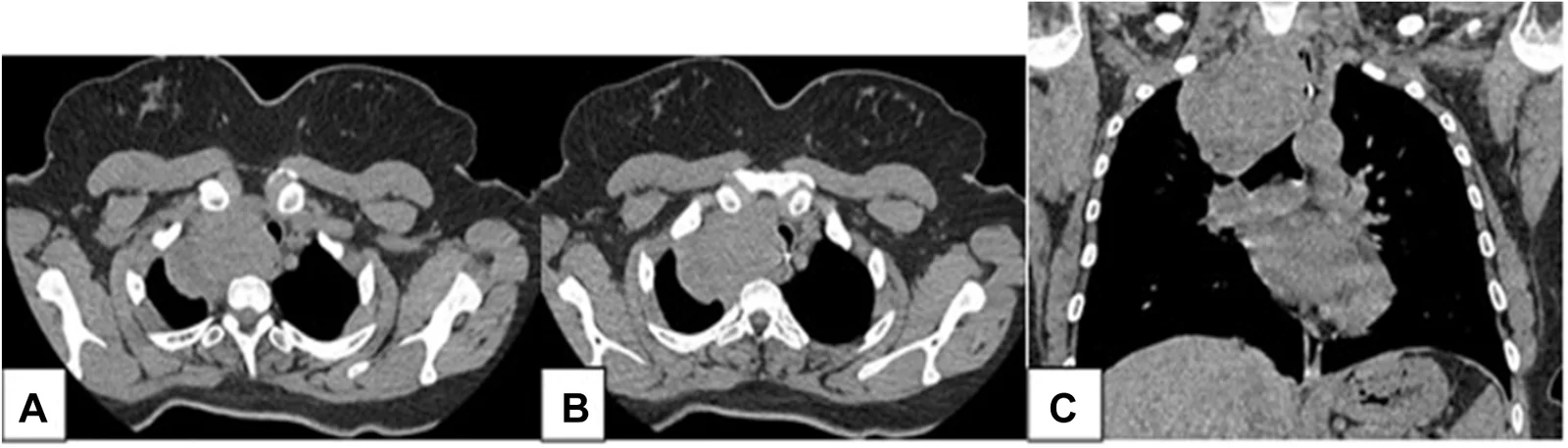
Noncontrast chest computed tomography (*CT*) scan at 1-month follow-up. Axial view **(A)** and **(B)** and coronal view **(C)** demonstrated a mass lesion compressing the trachea, with differential diagnoses including hematoma, substernal goiter, or pseudoaneurysm.

**Fig 3:**
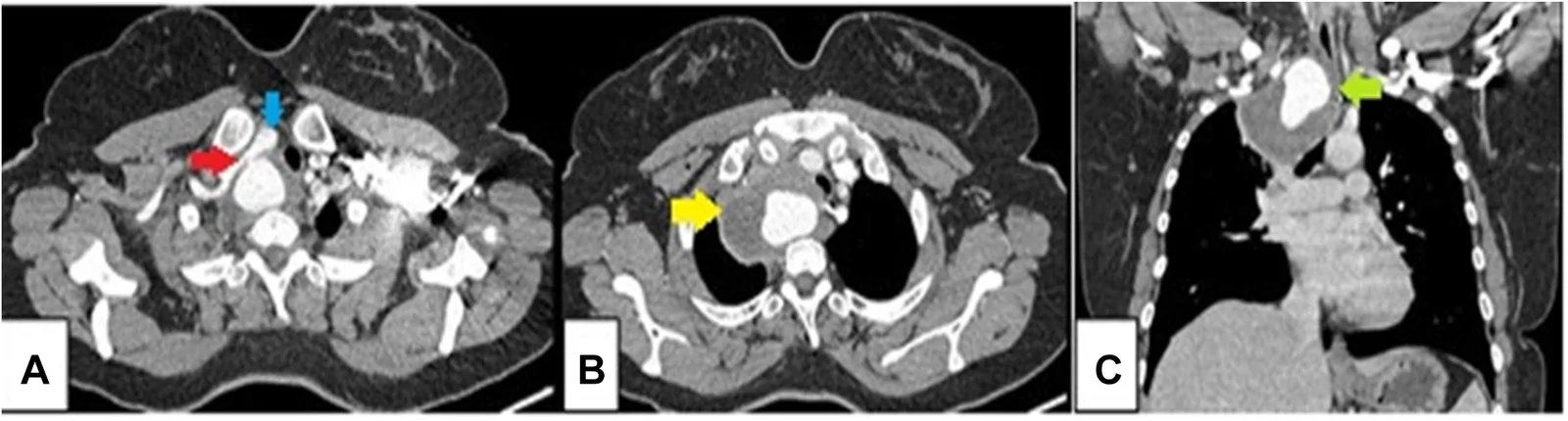
Computed tomography angiography (*CTA*). **A,** CTA shows the diameter of the right subclavian artery pseudoaneurysm, measuring 6 × 7 cm, compressing the trachea with deviation to the *left*. **B,** CTA image shows the pseudoaneurysm sac in the right supraclavicular region, demonstrating active blood leakage through a defect from the right subclavian artery. **C,** Coronal CTA image demonstrates the formation of a pseudoaneurysm arising from the right subclavian artery. Schematic overlay with colored arrows: *blue arrow* = brachiocephalic trunk at its bifurcation, *red arrow* = subclavian artery at the site of injury, *yellow arrow* = pseudoaneurysm sac, and *green arrow* = tracheal compression.

Surgical intervention was performed under general anesthesia through a partial median sternotomy extended obliquely to an 8-cm subclavicular incision. Vascular control was achieved proximally at the brachiocephalic trunk, right common carotid artery, and the origin of the right subclavian artery and distally beyond the pseudoaneurysm, including the vertebral artery. After excision of the pseudoaneurysm sac and debridement of the injured segment, the defect was repaired with a 7-mm Dacron interposition graft using end-to-end anastomoses (Fig 4).

**Fig 4:**
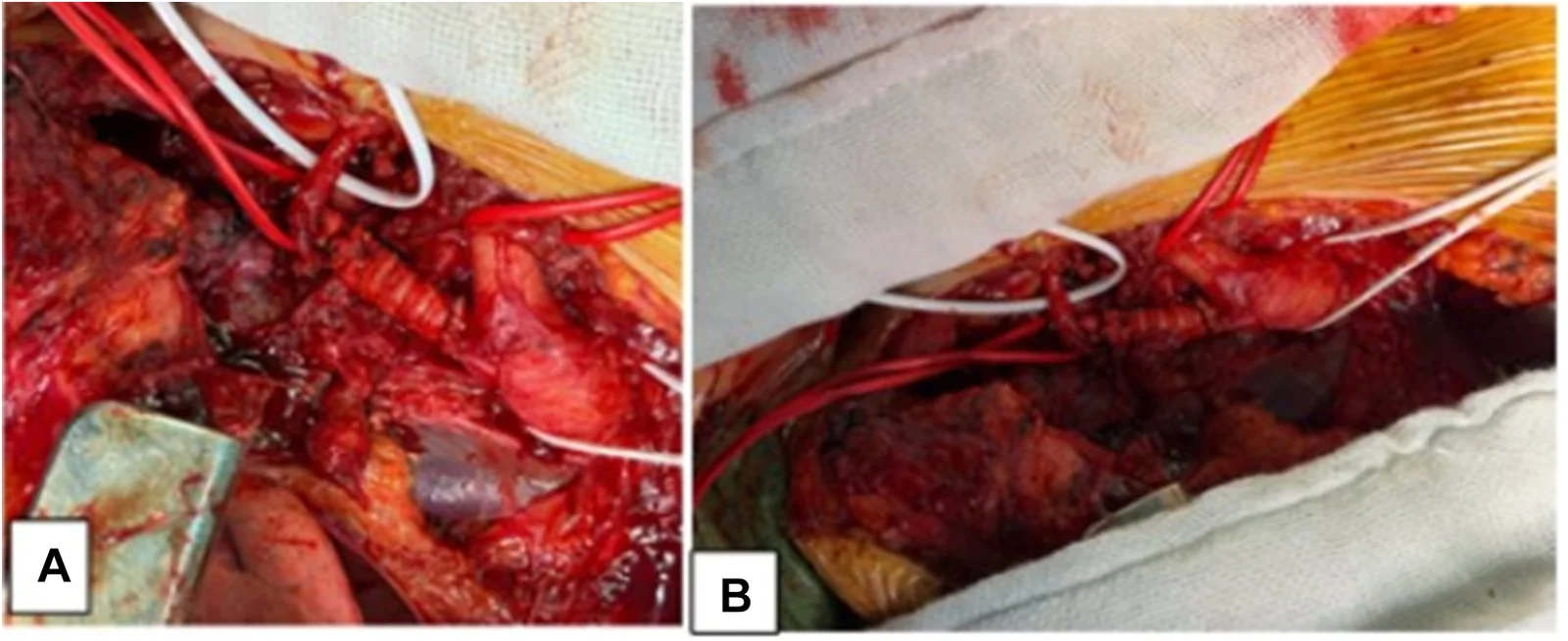
Surgical technique and direct repair. **A,** Intraoperative image showing secure vascular control (vascular loops around the brachiocephalic trunk, common carotid artery, and vertebral artery). **B,** Image after excision of the pseudoaneurysm sac and interposition of a 7-mm Dacron graft between the two ends of the right subclavian artery after resection of the injured segment.

The hospital stay lasted 3 days, with 24 hours in the intensive care unit for hemodynamic monitoring, followed by 2 days in the general ward without complications. Follow-up Doppler ultrasound confirmed complete graft patency, preservation of triphasic waveforms in the superficial and deep palmar arches of the right upper extremity, and complete resolution of hoarseness and dysphagia. Plain chest radiography at 1 and 3 months showed resolution of tracheal compression, and continued Doppler assessment demonstrated graft patency with sustained clinical improvement (Fig 5).

**Fig 5:**
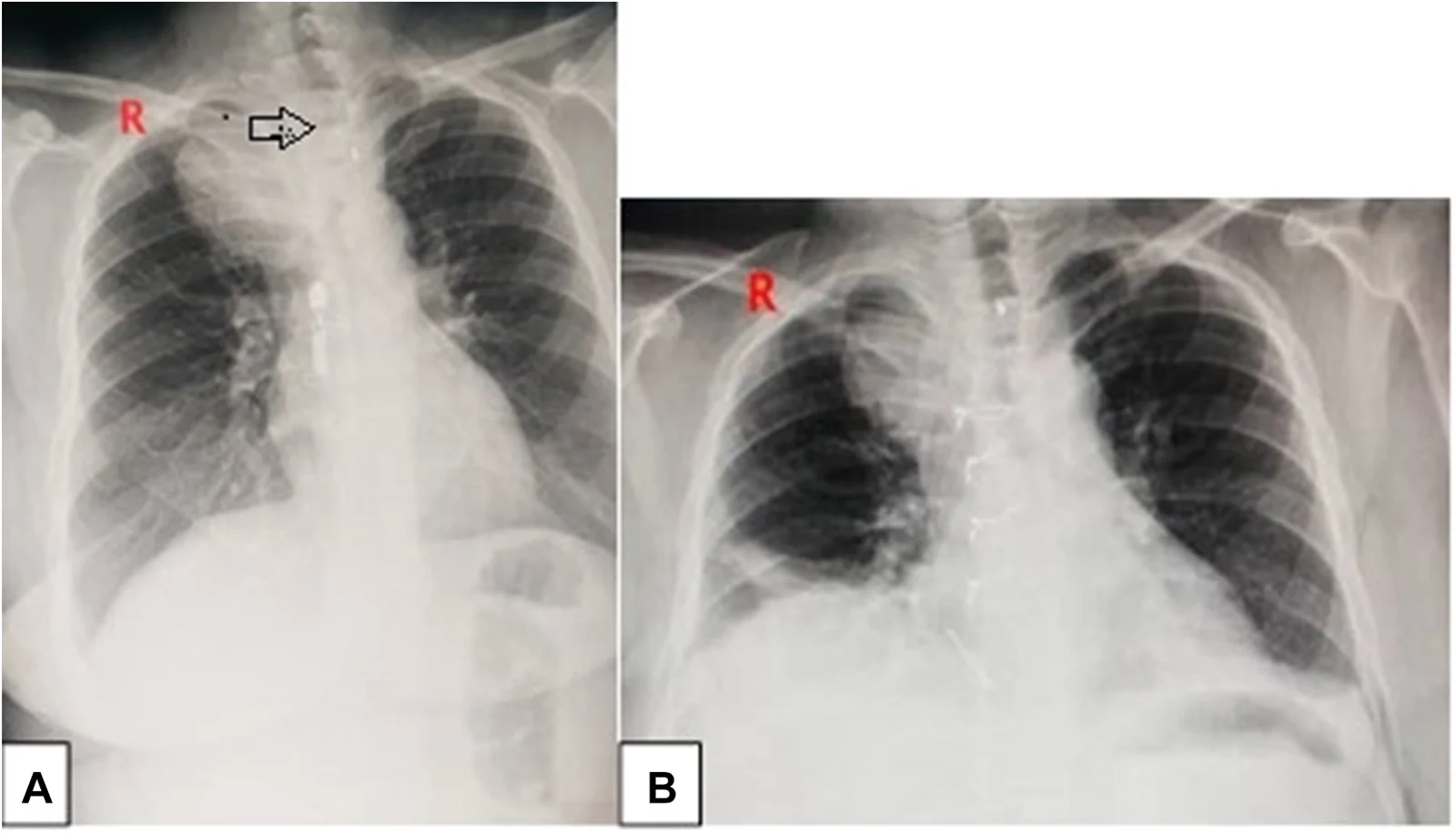
**A,** Plain chest X-ray before surgery showing the mass effect compressing the trachea (the *arrow* indicates tracheal deviation). **B,** Plain chest X-ray 3 months after surgery showing resolution of the tracheal compression and return to normal tracheal position, with sternal wires visible and the vascular graft not radiographically apparent.

## Discussion

Traumatic pseudoaneurysm of the subclavian artery is a rare condition, often presenting with delayed symptoms and a high risk of life-threatening complications.^1^^,^^3^^,^^5^ The present case of a 46-year-old female who developed a 6 × 7 cm pseudoaneurysm with 70% tracheal stenosis 1 month after a penetrating war shrapnel injury demonstrates successful direct surgical repair using a 7-mm Dacron interposition graft. This resulted in complete patency of the right subclavian artery, preserved triphasic signals in the palmar arches, and resolution of voice changes and swallowing difficulty during 3-month follow-up, with a short, uncomplicated hospital course.

This case differs from a previously reported 26-year-old male with blunt trauma and a closed clavicle fracture, in whom a 3-cm pseudoaneurysm was managed with a hybrid approach combining endovascular balloon occlusion for proximal control and open surgical bypass graft interposition. In that case, the presence of a clavicle fracture and associated bony constraints justified the use of balloon occlusion to facilitate exposure. In contrast, this case was characterized by a penetrating etiology (war shrapnel) with a substantially larger pseudoaneurysm measuring 6 × 7 cm (compared with 3 cm in the hybrid case), arising from an 8 × 9 mm arterial wall defect, and causing 70% tracheal stenosis because of acute airway compression. These features necessitated a direct surgical approach without endovascular assistance, as immediate decompression and complete sac excision were required.^2^

Early diagnosis has become feasible with CTA, now regarded as the gold-standard imaging modality, allowing accurate assessment of lesion size, progression, and relationships with adjacent structures, such as the vertebral artery.^6^^,^^7^ Contemporary literature increasingly favors endovascular stent grafting over open repair, citing high short-term technical success rates, reduced perioperative morbidity, and feasibility even in anatomically challenging proximal lesions.^6^^,^^8^

Endovascular repair was deemed unsuitable in this patient for several reasons. First, the presence of an acute surgical emergency because of airway compression and dyspnea required immediate decompression, which cannot be achieved rapidly with catheter-based techniques. Second, the close proximity of the pseudoaneurysm to the carotid artery origin and vertebral artery branching carried a risk of cerebral hypoperfusion or vertebral steal syndrome if stent migration or malposition occurred. Third, severe tracheal stenosis with 70% narrowing constituted an airway emergency requiring direct mass reduction. Fourth, in the setting of penetrating war shrapnel injury, irregular arterial walls and inflamed, scarred tissues decrease the predicted efficacy and durability of covered stents. Finally, at the time of presentation, the required endovascular devices and facilities were not available, making a percutaneous approach impractical.

In pseudoaneurysms, large sac size does not inherently increase the risk of endoleak because the lesion is contained by the adventitia and adjacent tissues; however, late stent failure from erosion, migration, or cyclic movement near the brachiocephalic trunk remains a concern.^6^^,^^7^ Open repair allowed complete resection of the pseudoaneurysm, precise control of the 8 × 9 mm defect, and secure anastomoses under direct visualization, thereby reducing the risk of delayed failure.^1^^,^^9^ This experience underscores the importance of individualized planning and the role of CTA surveillance in early detection.^5^

Although open surgical repair is considered the gold standard in complex cases such as this, it carries specific risks, particularly in the setting of war-related penetrating injuries. The surrounding tissues are often inflamed, scarred, and contaminated with metallic microparticles, which may increase the likelihood of complications.^8^^,^^10^ These include arterial thrombosis, which is rare but may require repeated thrombectomy or lifelong anticoagulation, especially in the presence of clavicle fracture or nonunion^3^; overall morbidity, as approximately one-third of cases are associated with chronic upper extremity neuropathy or brachial plexus injury, resulting in long-term functional disability^9^; mortality, reported between 10% and 35% in penetrating injuries and higher when presentation is delayed or associated with hemorrhagic shock and multiple injuries^1^^,^^11^; endoleak, reported in about 6.1% of endovascular repairs, sometimes requiring additional intervention or conversion to open surgery if sac enlargement persists^6^; and peripheral embolism, which may occur in about 0.4% of cases associated with clavicle fracture, resulting in upper-extremity ischemia or, rarely, cerebrovascular events.^3^ In this case, these complications were avoided through meticulous vascular control, appropriate sizing of the Dacron graft, intensive postoperative monitoring, and regular Doppler ultrasound follow-up. Nevertheless, patients who undergo repair of subclavian artery pseudoaneurysms following war shrapnel injuries remain at risk for late complications, underscoring the need for lifelong vascular surveillance.^10^

## Conclusions

This case demonstrates successful direct surgical repair of a traumatic pseudoaneurysm of the right subclavian artery with delayed presentation, large size (6 × 7 cm), and severe tracheal stenosis (70%) following a penetrating war shrapnel injury. Open surgical reconstruction achieved complete graft patency and symptom resolution without perioperative complications. This case highlights the importance of (1) repeated CTA surveillance after high-energy penetrating trauma even when initial imaging is negative, (2) individualized therapeutic decision-making based on anatomy and complications, and (3) lifelong follow-up to detect delayed graft-related complications.

## Consent

Written informed consent for publication of the case details and accompanying images was obtained from the patient. A copy of the consent form is available for review by the editor-in-chief upon request.

## Data availability

Not applicable. This article is a case report and does not contain original research data.

## Declaration of generative AI and AI-assisted technologies in the writing process

During the preparation of this work, the authors used ChatGPT (OpenAI) for spell-checking and grammatical correction only. After using this tool, the authors reviewed and edited the content as needed and take full responsibility for the content of the publication.

## Ethical considerations

This case report was conducted in accordance with the World Medical Association Declaration of Helsinki. The institutional review board of the authors' institution confirmed that ethical approval was not required for case reports at our institution. Patient confidentiality was maintained throughout.

## Funding

None.

## Disclosures

None.
